# Caregiver-child interaction as an effective tool for identifying autism spectrum disorder: evidence from EEG analysis

**DOI:** 10.1186/s13034-023-00690-z

**Published:** 2023-12-14

**Authors:** Lin Deng, Wei-zhong He, Qing-li Zhang, Ling Wei, Yuan Dai, Yu-qi Liu, Zi-lin Chen, Tai Ren, Lin-li Zhang, Jing-bo Gong, Fei Li

**Affiliations:** 1 Department of Developmental and Behavioral Pediatric and Child Primary Care & Ministry of Education, Shanghai Key Laboratory for Children’s Environmental Health, Xinhua Hospital, Shanghai Jiao Tong University School of Medicine, Shanghai, 200092 China; 2 Ministry of Education - Shanghai Key Laboratory of Children’s Environmental Health, Xinhua Hospital, Shanghai Jiao Tong University School of Medicine, Shanghai, 200092 China; 3 Shanghai Changning Mental Health Center, Shanghai, 200335 China; 4 Academy of Medical Engineering and Translational Medicine, Tianjin University, Tianjin, 300072 China; 5 College of Medical Imaging, Shanghai University of Medicine & Health Science, Shanghai, China

**Keywords:** Autism spectrum disorder, Caregiver-child interaction, Social interaction, Electroencephalography

## Abstract

**Background:**

Autism Spectrum Disorder (ASD) is a complex neurodevelopmental disorder that affects individuals across their lifespan. Early diagnosis and intervention are crucial for improving outcomes. However, current diagnostic methods are often time-consuming, and costly, making them inaccessible to many families. In the current study, we aim to test caregiver-child interaction as a potential tool for screening children with ASD in clinic.

**Methods:**

We enrolled 85 preschool children (Mean age: 4.90 ± 0.65 years, 70.6% male), including ASD children with or without developmental delay (DD), and typical development (TD) children, along with their caregivers. ASD core symptoms were evaluated by Childhood Autism Rating Scale (CARS) and Autism Diagnostic Observation Schedule-Calibrated Severity Scores (ADOS-CSS). Behavioral indicators were derived from video encoding of caregiver-child interaction, including social involvement of children (SIC), interaction time (IT), response of children to social cues (RSC), time for caregiver initiated social interactions (GIS) and time for children initiated social interactions (CIS)). Power spectral density (PSD) values were calculated by EEG signals simultaneously recorded. Partial Pearson correlation analysis was used in both ASD groups to investigate the correlation among behavioral indicators scores and ASD symptom severity and PSD values. Receiver operating characteristic (ROC) analysis was used to describe the discrimination accuracy of behavioral indicators.

**Results:**

Compared to TD group, both ASD groups demonstrated significant lower scores of SIC, IT, RSC, CIS (all *p* values < 0.05), and significant higher time for GIS (all *p* values < 0.01). SIC scores negatively correlated with CARS (*p* = 0.006) and ADOS-CSS (*p* = 0.023) in the ASD with DD group. Compared to TD group, PSD values elevated in ASD groups (all *p* values < 0.05), and was associated with SIC (theta band: *p* = 0.005; alpha band: *p* = 0.003) but not IQ levels. SIC was effective in identifying both ASD groups (sensitivity/specificity: ASD children with DD, 76.5%/66.7%; ASD children without DD, 82.6%/82.2%).

**Conclusion:**

Our results verified the behavioral paradigm of caregiver-child interaction as an efficient tool for early ASD screening.

**Supplementary Information:**

The online version contains supplementary material available at 10.1186/s13034-023-00690-z.

## Introduction

Autism spectrum disorder (ASD), a complex neurodevelopmental disorder with a current prevalence rate of 2.3% in the United States, is characterized by social skills deficits [1, 2]. Early identification is a crucial step in improving the prognosis [3] and ensuring timely access to early intervention strategies for children with ASD [4]. ASD diagnosis typically occurs at the age of five years [5]; however, signs of social abnormalities in autistic children [6], such as difficulty in perceiving and recognizing other people’s faces, emotional expressions [7], eyes [8], movements [9], and mental states [10], can manifest as early as infancy and impede their daily functioning. Current diagnosis of ASD is built on time-consuming assessment by specialized developmental-behavioral pediatrician. With 90% of people with ASD residing in low- and middle-income countries and regions, there is a critical need for low-cost screening tools that do not require trained professionals [11].

The widely used caregiver-reported measures for scanning ASD, like the Modified Checklist (M-CHAT) [12] have limitations in terms of accuracy despite being cost-effective, such as relying on subjective reporting, potentially introducing biases due to caregivers’ beliefs and experiences, as well as education differences [13]. Relying solely on screening questionnaires may therefore overlook subtle or nuanced symptoms and identify only the most apparent developmental and behavioral issues [14]. Furthermore, diagnostic scales such as Autism Diagnostic Observation Schedule (ADOS) [15] and Autism Diagnostic Interview-Revised (ADI-R) [16], require trained experts to conduct lengthy interviews, which limits their applicability given the high incidence of ASD. Therefore, screening assessments of social skills in children with ASD should focuse on real-life social situations, which would lead to a more objective and reliable identification of ASD and can be conducted in a cost-effective manner.

Caregiver-child interaction is an important foundation for children’s cognitive, linguistic, and socio-emotional development [17], and serves as a crucial starting point for acquiring interactive skills, including social communication skills [18, 19]. This interaction provides language and social stimuli that support the development of social skills [20], as caregivers provide feedback [21] on their children’s behavior to aid in their developmental process. As the most familiar social environment for children [22], caregiver-child interaction is applied in the early screening of ASD children to assess the performance of interaction and create conditions that maximize social interaction [23]. The content of caregiver-child interaction provides a direct source for clinician or other therapists to guide family intervention for ASD, making it an important tool for extensive early screening, diagnosis, and intervention of ASD children with significant health and economic implications. Therefore, this study aims to investigate the potential of caregiver-child interaction as an efficacious tool for early screening of children with ASD.

Advancements in electrophysiology tools have led to increased ecological validity of research on social interaction [24, 25], such as the use of EEG. EEG allows researchers to examine the natural electrical activity of the brain during different stimuli and conditions with high time resolution, portability, and tolerance to movement. Moreover, EEG signals reflect postsynaptic activity, while EEG power indicates the excitability of neuronal groups [26]. Studies [27–29] have demonstrated an association between behaviors observed during caregiver-infant interactions and infants’ EEG activity. A Bernier, SD Calkins and MA Bell [29] found that higher quality maternal behavior during mother-infant interactions predicted higher frontal alpha and theta resting EEG power at 10 and 24 months. Researchers have also found that children with autism show anomalies in EEG power spectrum from infancy, they exhibit higher alpha power and lower theta power for static faces relative to objects [30], in contrast to typical developmental infants [31]. LJ Gabard-Durnam, C Wilkinson, K Kapur, H Tager-Flusberg, AR Levin and CA Nelson [32]found that EEG power could consistently distinguish infants with ASD diagnoses from others. Therefore, this study employs EEG power spectrum to further support the reliability of the behavioral paradigm of caregiver-child interactions assessing ASD social interaction.

In this study, we utilized free play derived from the ADOS assessment as a caregiver-child interaction task to ensure natural face-to-face interactions between children with ASD and their caregivers. To account for variability in interactions, we employed micro-coding to identify participation status offline and calculate behavioral indicators based on it to quantify caregiver-child natural interactions. As ASD is a complex and heterogeneous clinical syndrome that includes individuals with varying levels of intellectual disability, language, and cognitive skills [2, 33], and individuals with higher cognitive skills may use scripted social behaviors to navigate social interactions [34, 35]. Thus, we plan to recruit preschool ASD children with different Intelligence qutient (IQ) levels and typically developing (TD) children, along with their caregivers, to engage in free play while simultaneously recording EEG and video signals. This will enable us to explore and evaluate effective indicators of atypical social patterns of ASD children and also investigate whether the IQ level of young children affects the social performance assessment of caregiver-child interaction.

Based on previous evidence, we hypothesize that:

1. The behavioral indicators of caregiver-child interactions can effectively differentiate between TD children and ASD children with varying levels of IQ.
2. Compared to TD children, ASD children have increased alpha power and theta power, and these PSD values are correlated with the behavioral indicators of caregiver-child interactions, regardless of IQ.

## Method

### Participants

85 children aged between 3 and 5 years were recruited from the Xinhua Hospital Affiliated to Shanghai Jiaotong University, including 40 ASD children (23 ASD children with developmental delay (DD), 17 ASD children without DD), and 45 typically developing (TD) children. All ASD children were evaluated and scored by experienced clinicians who specialize in identifying ASD, based on the Diagnostic and Statistical Manual of Mental Disorders (Fifth Edition, DSM-5). And ASD children scored above the threshold on both the Childhood Autism Rating Scale (CARS) and the Autism Diagnostic Observation Schedule (ADOS). Children with a history of neurological or genetic disorders, or those unable to engage in 3–5 min of interaction with a caregiver while wearing an EEG cap, were excluded from the study. TD children had no history of developmental disease and did not have any first-degree relatives with ASD. Caregivers of TD children were asked to complete the Chinese validated version of Social Responsiveness Scale (SRS) and only those who screened negative were included in this study. IQ of both ASD and TD children were assessed using the Wechsler Preschool and Primary Scale of Intelligence (WPPSI) [36]. The ASD children with DD group had Full Scale IQ (FSIQ) score at least two standard deviations below the average (FSIQ < 70), while the ASD children without DD group and TD children group had FSIQ score above 70. The study was conducted in accordance with the Declaration of Helsinki, with approval from the Ethical Committee of the Xinhua Hospital Affiliated to Shanghai Jiaotong University, and in compliance with all applicable laws and regulations. Written informed consent was obtained from the children’s caregivers. All necessary biosecurity and institutional safety protocols were followed during the study.

### Measures

#### Caregiver-child dyads coding

**Fig. 1:**
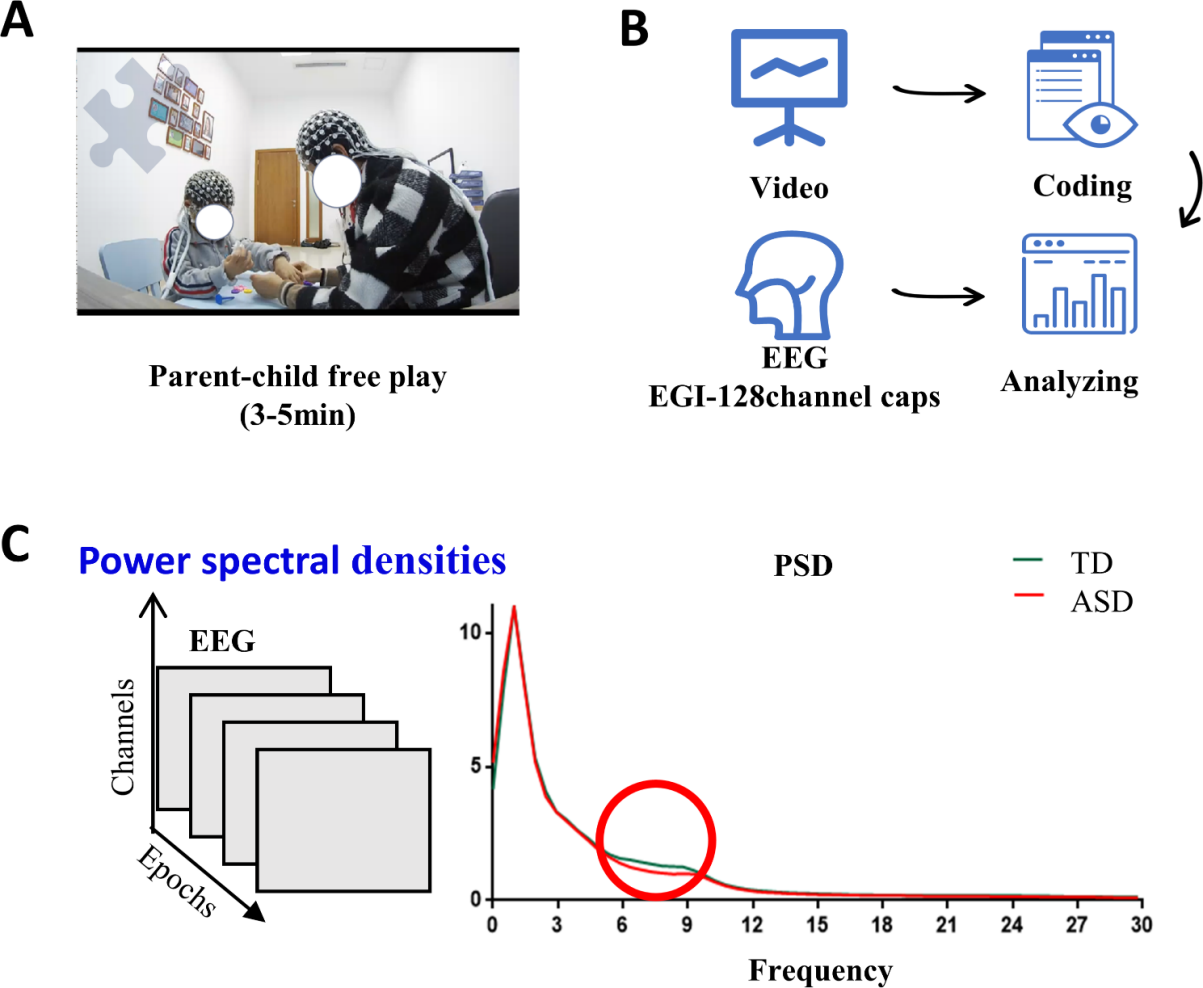
Study flowchart. (**A**) All participants engaged in a 3–5 min free play session face-to-face with their caregiver, while simultaneously collecting video and EEG signals. (**B**) After offline coding of the interaction video and preprocessing of EEG data, indicators were calculated and statistical analysis was performed. (**C**) After preprocessing the EEG data, PSD (power spectral density) values were obtained through FFT transformation in theta and alpha bands

To encourage natural and spontaneous interaction between the children and their most familiar caregivers, we provided a 3–5 min session of free play involving puzzles and blocks [37, 38]. Caregivers and children were seated at a table and wearing EEG caps. Their interaction was recorded by a camera while EEG was captured simultaneously (see Fig. 1A). Two researchers utilized the ELAN (EUDICO Language Annotator, version 6.2) [39] program allowing for stepping through the media with 1 s to identify and categorize social interaction between caregivers and children in the videos, according to a behavioral coding system described in detail in Supplemental Figure S1. Subsequently, five behavioral indicators were calculated based on the coding results for further analysis: Social Involvement of Children, Interaction Time, Response of Children to Social Cues, time for Caregiver Initiated Social interactions and time for Children Initiated Social interactions. Supplemental Table S1 provides definitions and examples for each code.

#### Behavior assessment

The ADOS is a widely utilized standardized diagnostic tool for ASD in both clinical and research settings [40] by evaluating social interaction, communication, and play in individuals with high-risk ASD. To account for variability in score across different modules of the ADOS, a mapping of ADOS module total scores to Calibrated Severity Scores (CSS) has been suggested [41]. The CSS system transforms the ADOS total score into a standardized score ranging from 1 to 10, with higher scores indicating greater severity of autistic features, based on the child’s actual age and language abilities. This standardized scoring system helps to provide a more accurate representation of the severity of autistic features in individuals with ASD.

The CARS [42] is a tool used to diagnose and assess the severity of ASD in children and consisted of 15 items rated on a 7-point scale from one to four; higher scores indicating a higher level of impairment. The CARS consists of three subscales [43]: Social Impairment (SI), Negative Emotionality (NE), and Distorted Sensory Response (DSR). The criterion validity for CARS with a cut-off of 30 resulted in sensitivity of 0.86 and specificity of 0.79 [44].

The SRS is commonly used to evaluate social deficits associated with ASD and other developmental disorders in clinical and research settings [45, 46]. The SRS provides a total score and scores on five subscales: social awareness, social cognition, social communication, social motivation, and autistic mannerisms. Multiple studies [47, 48] have reported high reliability and validity of the SRS, including the Chinese Mandarin version, which showed internal consistency for the total scale of 0.871–0.922, and test-retest reliability of 0.81–0.94.

#### EEG recording and pre-processing

During a 3–5 min free play session between caregivers and children, we recorded EEG signals using a high-density 128-channel Electrical Geodesics, Inc (EGI) system with a vertex reference (channel Cz) and a sampling rate of 1000 Hz. To ensure high-performance data, we kept impedances below 100KΩ [32]. Despite we recorded both child and their caregiver’s EEG, the physical movements necessitated by taking care of children led to an increased presence of motion artifacts in caregivers’ EEG data, rendering it unsuitable for further analysis. Thus, in this study, we focus exclusively on analyzing and discussing the EEG signals of children. MATLAB [49] and the EEGLab [50] toolbox were used to process offline. A bandpass filter between 0.5 and 45 Hz and a 50 Hz notch filter were consistently applied to the continuous EEG data. We retained 82 channels for analysis after excluding 46 peripheral “skirt channels“ [51] from EEG data that are particularly sensitive to noise and muscle artifact to reduce noise and muscle artifact, and interpolated any noisy electrodes. All segments were visually inspected, and those containing myoelectricity or other artefacts not related to blinks were manually removed. Afterward, 1-s epoch segments were created from the preprocessed EEG data. Independent component analysis (ICA) [50] was used to identify and eliminate eye blink, movement, and muscle activity artifacts after physically confirming artifacts rejection by visual examination. Prior to spectral analysis, the data was re-referenced to the average of the mastoids.

#### Alpha and theta Power Spectral Density (PSD)

The power spectral density (PSD) of the theta (4–7 Hz) and alpha (8–13 Hz) frequency bands are computed by applying the fast FFT algorithm, squaring the resulting signal to obtain amplitude, transforming the bilateral spectrum into a unilateral spectrum, and dividing it by the frequency resolution. This gave a single estimation of PSD at each of 82 channels.

### Statistical analysis

The statistical analyses employed SPSS software, version 23, with the Wilcoxon rank sum test and Analysis of Variance (ANOVA) used to compare mean ± standard error of continuous variables between the ASD and TD groups. Significance level (α) was set at 0.05. Prior to analysis, behavioral data and PSD values were transformed by square root to meet normal distributional assumptions. General linear model (GLM) was used to compare groups on EEG PSD.

The application of Receiver Operating Characteristics (ROC) [52] analysis was implemented with the intent of computing the Area Under the Curve (AUC) [53], serving as a metric for the discriminative capability of behavioral indicators in distinguishing between ASD groups and TD group. The ROC analysis, offering sensitivity and 1 - specificity data for a range of thresholds, aids in choosing the best potential cutoff for each separate group comparison. We also examined the accuracy, sensitivity, and specificity of these behavioral indicators in ASD groups (ASD children with and without DD) and the TD group.

A series of partial Pearson correlation analyses were conducted to evaluate the dimensional relationships among EEG PSD and IQ level and behavioral indicators (social involvement of children, Interaction Time, Response of Children to Social Cues, time for Caregiver Initiated Social interactions, time for Children Initiated Social interactions) in all groups. All the *p* values are adjusted for multiple comparison (Bonferroni).

## Results

### Participant information

The demographic and clinical characteristics of the three groups were shown in Table 1. The average age of participants was 4.87 (0.18) years in ASD children with DD group, 4.80 (0.26) years in ASD children without DD, and 4.98 (0.15) years in TD children group. There were no significant differences in age among the three groups. The ASD children with DD group (82.6%, *p* = 0.036) and the ASD children without DD group (94.1%, *p* < 0.001) had a significant higher proportion of male participants than TD children group (51.1%). Both verbal (*p* < 0.001) and performance IQ (*p* < 0.001) were significantly lower in the ASD children with DD group than in the other two groups. After adjusting for the effect of sex, there were no differences in symptom severity scores between ASD children with DD group and ASD children without DD group (ADOS-CSS, *p* = 0.875; CARS, *p* = 0.123; SRS, *p* = 0.159).

**Table 1:** The demographic and clinical (mean (SE)) characteristics of three groups

	ASD children with DD group	ASD children without DD group	TD children group	F-Value	*P*
N	23	17	45		
Female (%)	4 (17.4)	1 (5.9)	22 (48.9)	6.906	0.002^ab^
Age	4.87 (0.18)	4.80 (0.26)	4.98 (0.15)	0.692	0.504
P_IQ	55.13 (1.91)	85.88 (3.74)	87.53 (3.92)	30.311	< 0.001^ac^
V_IQ	47.91 (1.38)	67.12 (4.85)	77.65 (2.41)	31.117	< 0.001^ac^
FSIQ	48.52 (1.33)	82.22 (4.55)	91.30 (2.26)	112.132	< 0.001^ac^
ADOS-CSS	6.96 (0.45)	7.06 (0.47)		-0.158	0.875
SRS	93.22 (4.86)	85.31 (4.80)		2.061	0.159
Social awareness	11.13 (0.58)	10.88 (0.46)		0.103	0.750
Social cognition	19.17 (1.14)	16.62 (0.76)		2.846	0.100
Social communication	32.96 (1.64)	30.50 (1.88)		0.953	0.335
Social motivation	15.00 (0.97)	13.88 (0.89)		0.666	0.420
Autistic mannerisms	14.96 (1.38)	13.44 (1.42)		0.559	0.459
CARS	35.87 (3.67)	34.15 (3.04)		2.485	0.123
Social Impairment	23.46 (0.66)	22.63 (1.63)		0.305	0.584
Negative Emotionality	6.63 (0.187)	6.19 (0.452)		1.439	0.238
Distorted Sensory Response	6.89 (0.26)	6.75 (0.50)		0.038	0.847

Notes: Values for age and all clinical test scores are presented as mean (SE). Abbreviations: ASD, autism spectrum disorder; DD, developmental delay; TD, typically developing; P_IQ, performance Intelligence Quotient; V_IQ, verbal Intelligence Quotient; FSIQ, full scale Intelligence Quotient; ADOS-CSS, Autism Diagnostic Observation Schedule- Calibrated Severity Score; SRS, Social Responsiveness Scale; CARS, Childhood Autism Rating Scale. ^a^, Significant difference between ASD children with DD group and TD group. ^b^, Significant difference between ASD children without DD group and TD group. ^c^, Significant difference between ASD children with DD group and ASD children without DD group. All p values are adjusted for multiple comparison (Bonferroni)

### Behavioral indicators and the associations with ASD symptoms

**Fig. 2:**
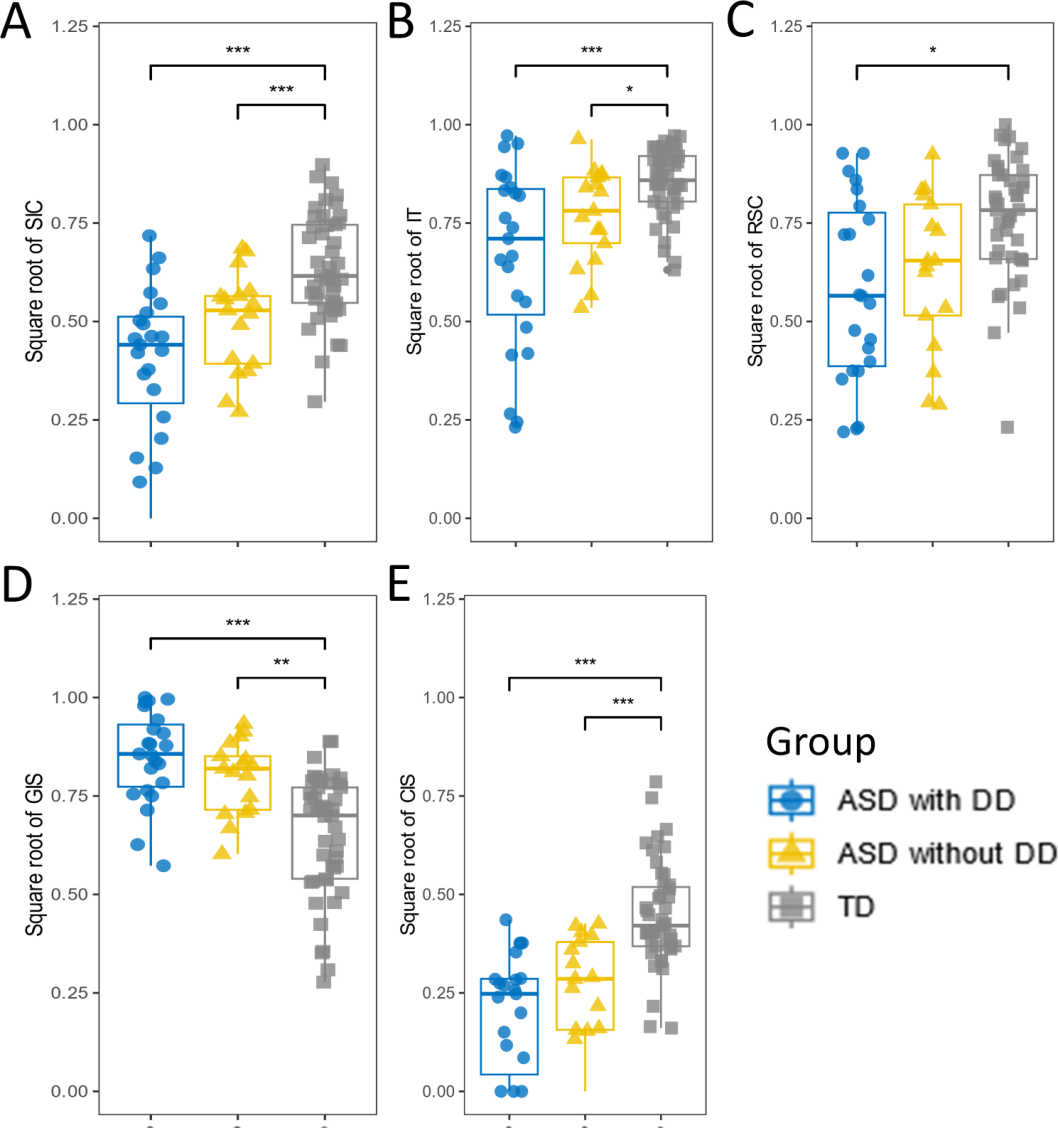
Comparison of behavioral coding indicators among ASD with DD, ASD without DD, and TD children. (**A**) Differences in Social Involvement of Children (SIC) among the three groups. (**B**) Differences in Interaction Time (IT) among the three groups. (**C**) Differences in Responding to Social Cues (RSC) among the three groups. (**D**) Differences in caregivers Initiated Social interaction (GIS) among the three groups. (**E**) Differences in children Initiated Social interaction (CIS) among the three groups. The standard error of the mean is displayed as error bars. Significant differences between groups are indicated by horizontal bars and statistical significance is denoted by asterisks (*, *p* < 0.05; **, *p* < 0.01; ***, *p* < 0.001). ASD with DD, Autism disorder spectrum children with development delay group; ASD without DD, Autism disorder spectrum children without development delay group; TD, typical development children group

Figure 2 presents a comparison of caregiver-child interaction performance among the groups, after sex adjustment. As shown in Table 2, compared to TD group, both ASD groups demonstrated significantly lower scores of the Social Involvement of Children, Interaction Time and Children Initiated Social interactions (all *p* values < 0.05), and significantly higher time for Caregiver Initiated Social interactions (all *p* values < 0.01). Only ASD children with DD group demonstrated a significant decrease in Response of Children to Social Cues (*p* = 0.01) compared to the TD children group.

**Table 2:** The behavioral indictors and PSD values (mean (SE)) of three groups

Indicators	Group	Mean (SE)	sex-adjusted mean	Bonferroni -*P* value
SIC	ASD with DD	0.424(0.031)	-0.214^a^	< 0.001
	ASD without DD	0.467(0.048)	-0.170^b^	0.001
	TD	0.638(0.020)		
IT	ASD with DD	0.694(0.036)	-0.161^a^	< 0.001
	ASD without DD	0.729(0.056)	-0.126^b^	0.021
	TD	0.855(0.013)		
RSC	ASD with DD	0.569(0.043)	-0.176^a^	0.010
	ASD without DD	0.640(0.056)	-0.111^b^	0.289
	TD	0.730(0.033)		
GIS	ASD with DD	0.850(0.017)	0.195^a^	< 0.001
	ASD without DD	0.797(0.033)	0.142^b^	0.003
	TD	0.650(0.023)		
CIS	ASD with DD	0.212(0.029)	-0.225^a^	< 0.001
	ASD without DD	0.241(0.038)	-0.195^b^	< 0.001
	TD	0.439(0.022)		
Theta PSD	ASD with DD	1.730(0.102)	0.310 ^a^	0.016
	ASD without DD	1.741(0.107)	0.309 ^b^	0.041
	TD	1.394(0.506)		
Alpha PSD	ASD with DD	1.214(0.065)	0.218 ^a^	0.012
	ASD without DD	1.178(0.742)	0.173 ^b^	0.122
	TD	0.973(0.036)		

Notes: Abbreviations: sex-adjusted mean, a sex-adjusted mean difference; ASD without DD, children with autism spectrum disorder without developmental delay; ASD with DD, children with autism spectrum disorder with developmental delay; TD, typically development; SIC, Social Involvement of Children; IT, Interaction Time; RSC, Response of children to Social Cues; GIS, Caregiver Initiated Social interaction; CIS, Children Initiated Social interactions. ^a^, sex-adjusted mean between ASD children with DD group and TD group; b, sex-adjusted mean between ASD children without DD group and TD group; All p values are adjusted for multiple comparison (Bonferroni). We investigated whether the behavioral indicators were associated with severity scores among autistic children after adjusting for sex (see Fig. 3 and Table S2, Bonferroni’s *p* = 0.005). The Social Involvement of Children (ASD children with DD group, *p* = 0.004; ASD children without DD group, *p* = 0.005), Interaction Time (ASD children with DD group, *p* = 0.001; ASD children without DD group, *p* = 0.003) and time for Caregiver Initiated Social interactions (ASD children with DD group, *p* = 0.004; ASD children without DD group, *p* = 0.002) showed significant correlations with the total score of CARS in both ASD groups. But Response of Children to Social Cues (*p* = 0.003) was only significant correlated with the total score of CARS in ASD children with DD group. Additionally, in ASD children with DD group, Social Involvement of Children was not only significantly correlated with CARS total scores (*p* = 0.004) but also correlated with ADOS-CSS score (although not corrected by Bonferroni, *p* = 0.020)

**Fig. 3:**
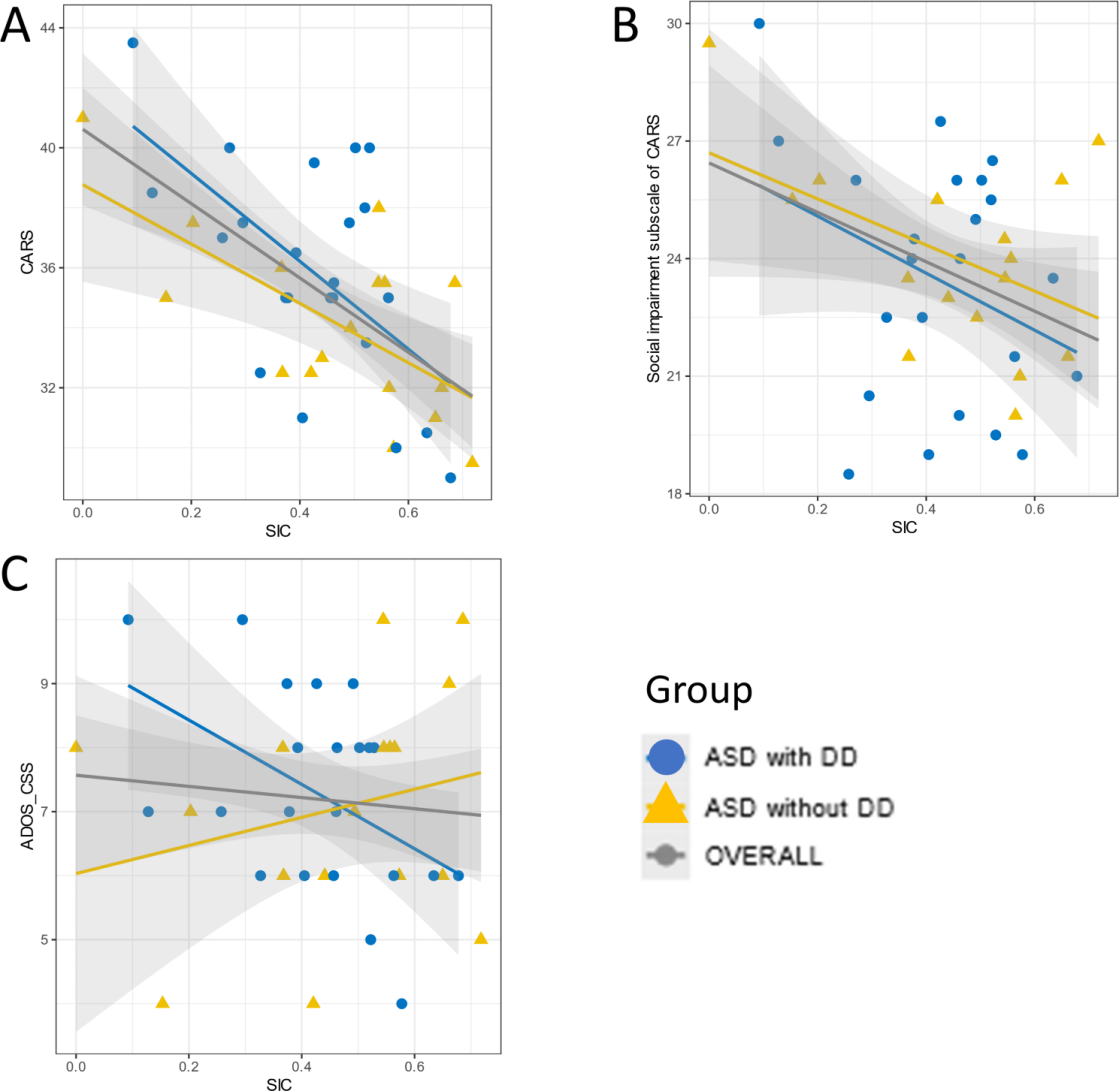
Scatterplots depicting the associations between symptom severity and SIC within each group. (**A**) Correlations between SIC and Childhood Autism Rating Scale (CARS) (**B**) Correlations between SIC and the social impairment subscale of CARS. (**C**) The correlations between SIC and the Autism Diagnostic Observation Schedule- Calibrated Severity Score (ADOS-CSS). For test statistics, see Table S2. ASD with DD, Autism disorder spectrum children with development delay group; ASD without DD, Autism disorder spectrum children without development delay group; TD, typical development children group; OVERALL, full sample

In our analysis, only male children with ASD exhibited significant correlations between all behavioral indicators, except Children Initiated Social interactions, and CARS scores (all *p* values < 0.001, Bonferroni corrected *p* < 0.005) after adjusting for verbal IQ when analyzing different sexes separately, as detailed in Table S2.

### The ROC analysis for behavioral indicators

**Fig. 4:**
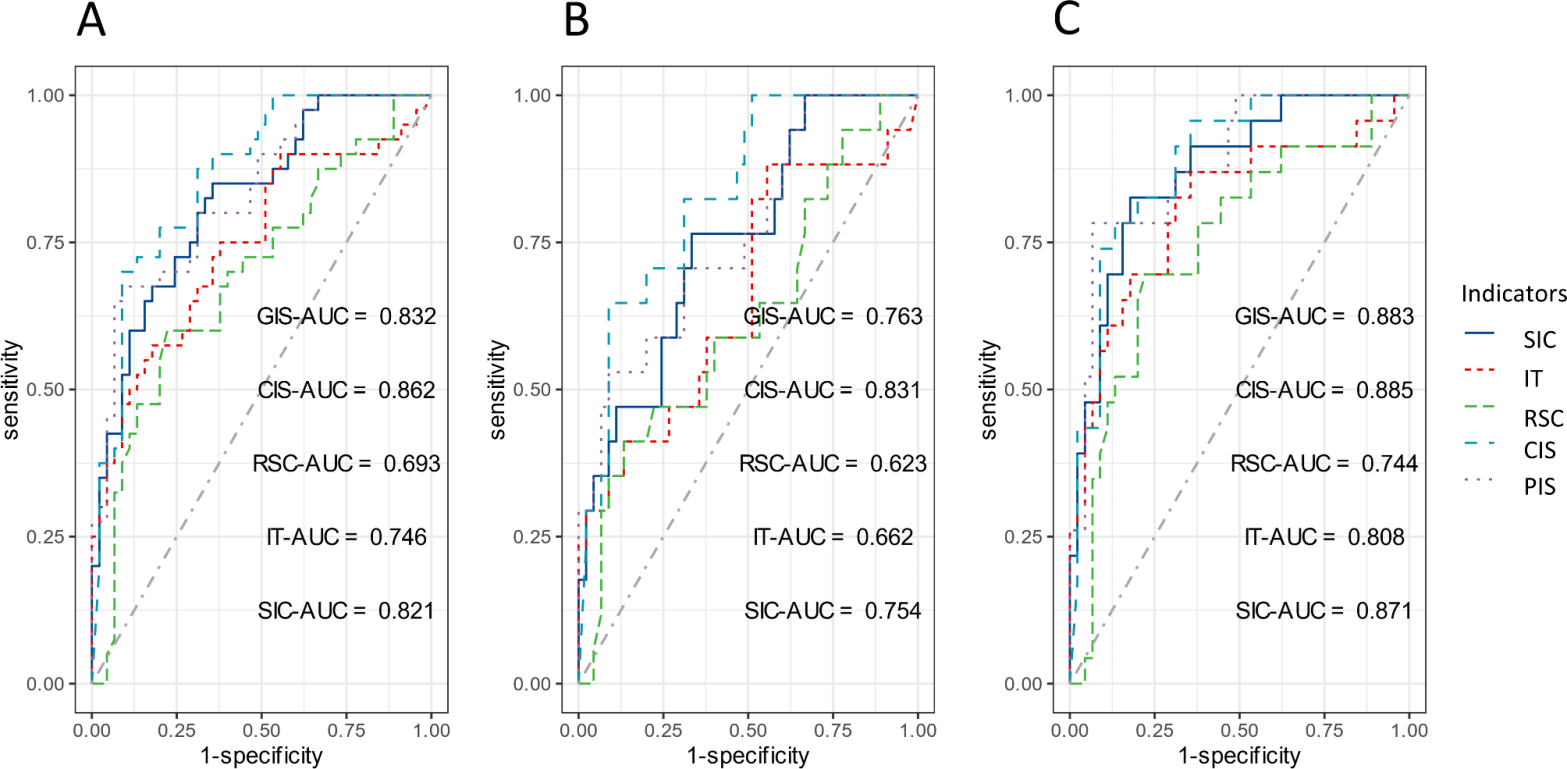
The differences in AUC analyze of behavioral indicators and PSD values in 3 groups. (**A**) The ROC curve of 5 behavioral indicators differentiating ASD children (with or without DD) from TD children. (**B**) The ROC curve of 5 behavioral indicators differentiating ASD children without DD from TD children. (**C**) The ROC curve of 5 behavioral indicators differentiating ASD children with DD from TD children. Abbreviations: ROC, receiver operating characteristic; AUC, area under the receiver operating curve; ASD, autism spectrum disorder; DD, developmental delay; TD, typically development; SIC, Social Involvement of Children; IT, Interaction Time; RSC, Response of children to Social Cues; GIS, Caregiver Initiated Social interaction; CIS, Children Initiated Social interactions

The ROC for 5 behavioral indicators for each of the 3 groups were shown in Table 3. Children Initiated Social interactions (sensitivity = 70%, specificity = 91.1%) was most effective in the full samples. Although Interaction Time (sensitivity = 88.2%) and Response of Children to Social Cues (sensitivity = 87.0%) are the most sensitive indicators in differentiating ASD children without/ with DD and TD, their specificity was low (Interaction Time, specificity = 44.4%; Response of Children to Social Cues, specificity = 46.7%). Social involvement of children (ASD children with DD, sensitivity = 76.5%; ASD children without DD, sensitivity = 82.6%) has the second highest sensitivity in identifying both groups of ASD children, and the specificity (ASD children with DD, specificity = 66.7%; ASD children without DD, specificity = 82.2%) is higher than both of the above two indicators (see Fig. 4).

**Table 3:** Results of AUC analyses in 3 groups

	Indicators	Sensitivity	Specificity	Accuracy
Full samples (*N* = 85)	SIC	67.5%	82.2%	82.1%
	IT	57.5%	82.2%	74.6%
	RSC	60.0%	77.8%	77.8%
	CIS	70.0%	91.1%	86.2%
	GIS	67.5%	91.1%	83.2%
ASD children without DD group and TD children group (*N* = 62)	SIC	76.5%	66.7%	75.4%
	IT	88.2%	44.4%	66.2%
	RSC	41.2%	86.7%	62.3%
	CIS	64.7%	91.1%	83.1%
	GIS	52.9%	91.1%	76.3%
ASD children with DD group and TD children group (*N* = 68)	SIC	82.6%	82.2%	87.1%
	IT	69.6%	82.2%	80.8%
	RSC	87.0%	46.7%	74.4%
	CIS	78.3%	86.7%	88.5%
	GIS	78.3%	93.3%	88.3%

Notes: Abbreviations: ASD, autism spectrum disorder; DD, developmental delay; TD, typically development; SIC, Social Involvement of Children; IT, Interaction Time; RSC, Response of children to Social Cues; GIS, Caregiver Initiated Social interaction; CIS, Children Initiated Social interactions

### EEG Power and its correlations with behavioral indicators and IQ level

**Fig. 5:**
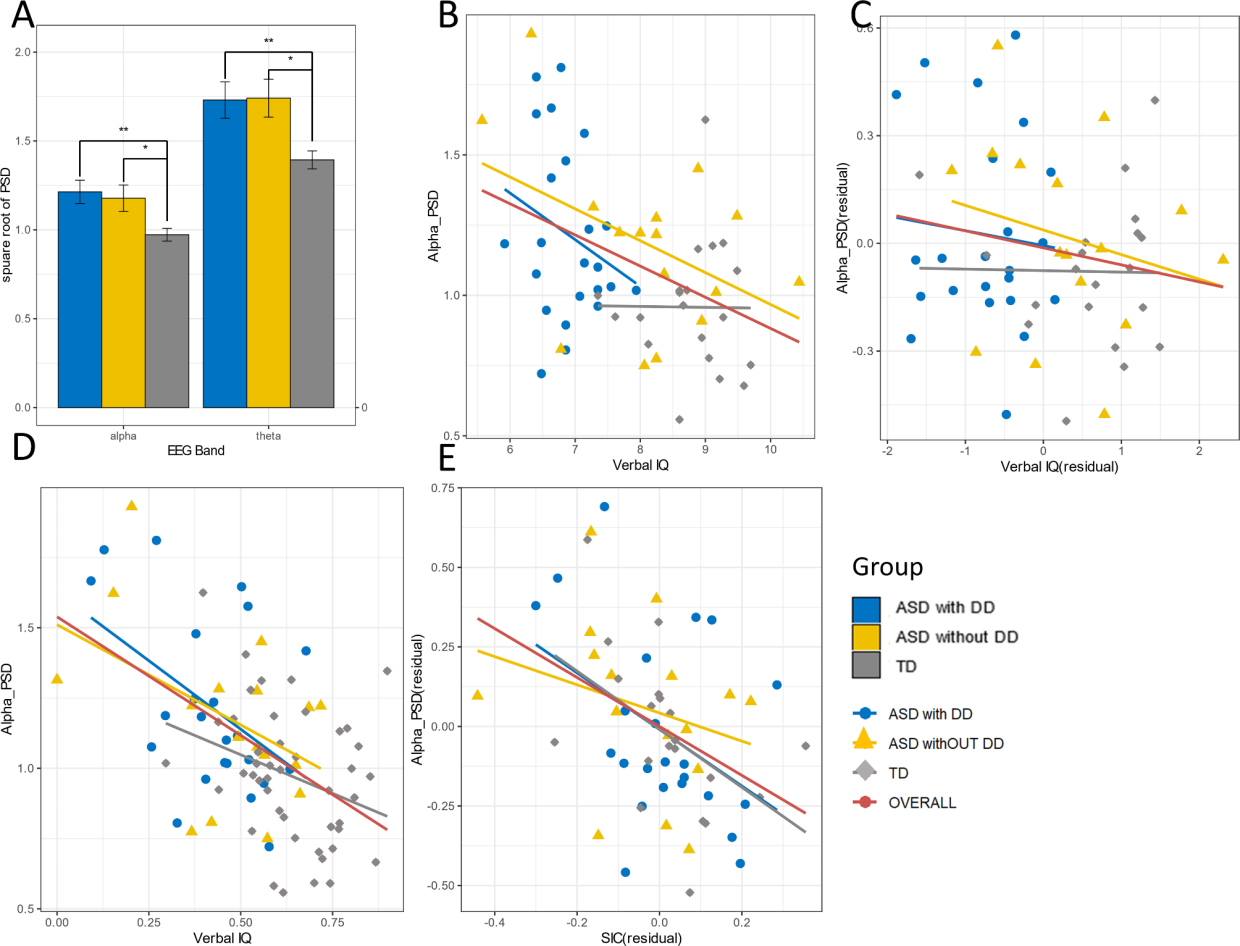
The differences in alpha PSD between the ASD and TD groups. (**A**) The alpha PSD of children in three groups. (**B**) Correlations between verbal IQ and PSD. (**C**) Correlations between verbal IQ and PSD residuals following partial SIC (i.e., correlation between residuals). (**D**) Correlation between the SIC and alpha band’s PSD. (**E**) Correlation between the residuals for the SIC and alpha PSD following the partial elimination of verbal IQ (i.e., correlation between residuals). Within each diagnostic group and for the whole sample, correlation lines are displayed. For test statistics, see Table 3. The standard error of the mean is displayed as error bars. Significant changes between conditions are indicated by horizontal bars and * (*, *p* < 0.05; **, *p* < 0.01; ***, *p* < 0.001. The p values weren’t adjusted for multiple comparison (Bonferroni)). ASD with DD, Autism disorder spectrum children with development delay group; ASD without DD, Autism disorder spectrum children without development delay group; TD, typical development children group

PSD was calculated for the theta and alpha bands in three groups. Channel-to-channel comparisons were conducted between the groups (see Fig. 5). PSD in both bands increased in ASD with DD group (alpha *p* = 0.012, theta *p* = 0.016) and only theta PSD increased in ASD without DD group (*p* = 0.041) than TD children group after adjusting for sex (see Table 2).

We investigated the association between PSD in the theta and alpha bands and behavioral indicators while adjusting for verbal IQ levels and sex in the full sample (performance IQ did not correlate with PSD values across all groups). Details were shown in Table 4. Specifically, we observed statistically significant correlations (Bonferroni-all *p* values < 0.005) between all the five behavioral indicators, IQ and the PSD values of alpha and theta band. Notably, these correlations remained significant even after adjusting for verbal IQ and sex. Among these indicators, the strongest correlation with PSD was Social Involvement of Children (Alpha, *p* = 0.001, Theta, *p* = 0.002), the second strongest correlation was Interaction Time (Alpha, *p* = 0.001, Theta, *p* = 0.002), and Caregiver Initiated Social interactions only significantly correlated with PSD in alpha band (*p* = 0.002) (see Table 4).

**Table 4:** Results of correlation analyses in full sample

	Alpha PSD		Theta PSD	
	Partial Pearson correlation, controlled for sex	Partial Pearson correlation, controlled for sex and Verbal IQ	Partial Pearson correlation, controlled for sex	Partial Pearson correlation, controlled for sex and Verbal IQ
SIC				
r	-0.493^a^	-0.414^a^	-0.450^a^	-0.410^a^
*p*	< 0.001	0.001	< 0.001	0.002
IT				
r	-0.476 ^a^	-0.419 ^a^	-0.474 ^a^	-0.401 ^a^
*p*	< 0.001	0.001	< 0.001	0.002
RSC				
r	-0.327 ^a^	-0.200	-0.331 ^a^	-0.194
*p*	0.002	0.137	0.002	0.148
CIS				
r	-0.376^a^	-0.269	-0.348^a^	-0.279
*p*	< 0.001	0.043	0.001	0.035
GIS				
r	0.419 ^a^	0.404 ^a^	0.356 ^a^	0.357
*p*	< 0.001	0.002	0.001	0.006

Notes: r presented as Pearson’s correlation coefficients, *p*, p value. Abbreviations: PSD, Power Spectral Density; IQ, Intelligence Quotient; SIC, Social involvement of the child; IT, Interaction Time; RSC, Response of children to Social Cues; GIS, Caregiver Initiated Social interaction; CIS, Children Initiated Social interactions. ^a^, Bonferroni’s correction for multiple correlations: *p* < 0.005

We only observed the associations between Interaction Time and both of the PSD values in males within the ASD group. Despite the correlation decreased after verbal IQ adjustment, it was noticeably stronger in males than female. What’s more, the associations between verbal IQ, SIC, and PSD in both theta and alpha band were stronger in males of TD children (as shown in Table S2).

## Discussion

In this study, we introduced a time-efficient and low-cost screening tool for ASD that does not require trained professionals. We quantify caregiver-child natural interactions via video-encoded behavioral indicators and employ EEG power spectrum analysis to further validate the reliability of these behavioral indicators. The scores of behavioral indicators of both ASD groups were lower than TD group and Social Involvement of Children is the most effective indicator in screening ASD children. And significantly higher PSD values were shown in ASD group, and were strongly correlated with behavioral indicators.

The first main finding was that, consistent with our first hypothesis, ASD children exhibited decreased levels in most of behavioral indicators, including dyadic interaction (Interaction Time), participation to social cues (Social Involvement of Children), initiation (Children Initiated Social interactions), and responsiveness to social cues (Response of Children to Social Cues) during caregiver-child interaction tasks, regardless of intellectual disability. Furthermore, the more severe the social impairment symptoms were, the lower levels of the child’s initiation of social interactions, response to social cues, and engagement with social cues were.

For the ROC, Interaction Time and Response of Children to Social Cues respectively exhibited the highest sensitivity in identifying both ASD groups, however, they presented a specificity under 50%. This may significantly elevate the risk of misdiagnosis [52, 53]. Conversely, though Social Involvement of Children though second in sensitivity, it offered a higher specificity than the two highest sensitivity indicators. These findings suggested that Social Involvement of Children may be a more consistent indicator of social deficits in children with ASD, irrespective of their IQ levels. Social Involvement of Children reflects both level of active social initiation during caregiver-child interaction and response behaviors to social cues initiated by caregivers. Previous studies in high-risk ASD population (e.g., ASD’s siblings) also highlighted the synchrony and infant/maternal responsiveness computed by frequency and duration of gaze, positive affect and vocalizations during infant-mother interaction can help predict the outcome of autism [54, 55].

We also found that, compared to the caregivers of TD children, the caregivers of ASD children had increased Caregiver Initiated Social interactions, indicating they initiated social interaction more urgently and frequently. Because parents’ hopes and expectations for their one and only child were so high—based on the fact that families in China tended to have only one child [56]. They are more desperately eager to witness progress in their child’s social interactions [57] and, as such, they invest more attention in social scenarios to avoid missing any subtle improvements. Caregivers aim to showcase their child’s optimal social performance to receive positive feedback.

Our results demonstrated sex difference in the associations between the behavioral indicators and the severity of autism symptoms, which was only found among male, and remained significant after adjusted verbal IQ. It is well established that autistic girls demonstrate higher levels of social motivation than autistic boys, increasing their opportunities for engaging social interaction [58], girls with ASD used compensatory behaviors, which appeared to mask their social challenges [59, 60]. Therefore, simple social tasks (free play with their caregivers) may be too simple to reflect their social deficiencies.

The second main finding in the present study was that, compared to TD group, significant increase in EEG PSD of alpha and theta power was observed in both ASD groups (except alpha band PSD in ASD without DD group, not corrected by Bonferroni) during caregiver-child interaction. Increased alpha and theta PSD values during caregiver-children interaction indicate that ASD children have atypical neural responses to social interaction [61, 62]. Some researchers [63, 64] proposed that this atypical neural activity may contribute to the atypical social impairments observed in ASD, as it may reflect a decreased ability to process and respond to social cues effectively [65]. Higher alpha power is in response to social stimuli such as faces and emotions in ASD children group, compared to TD children and positively associated with autistic trait expression [66–68]. And higher theta activity in ASD may reflect difficulties in integrating information from multiple sources and potentially result in inadequate processing and interpretation of social cues [69]. Although alpha band PSD in the ASD without DD group did not withstand Bonferroni correction, this could be attributed to the relatively smaller sample size of this group than the two other groups, leading to increased individual heterogeneity in alpha power spectrum. In future research, we validate our results by expanding the sample size.

The negative correlations between PSD of alpha and theta band values and behavioral indicators, particularly Social Involvement of Children, were shown in both ASD groups. Our findings reinforce that the reduced initiation and lower responsiveness to social cues among children with ASD correlate with increases in alpha and theta band EEG power. This process may involve attentional processes, motor imitation [70] during the interaction in ASD, such as decreased focus on social cues, faces and using less gestures [61, 62, 66–68]. Our study underscores the significance of considering children’s real-world social interaction behavior in identifying and diagnosing ASD.

Furthermore, significant correlations between behavioral indicators and PSD values were even persistent after controlling for verbal IQ and sex differences. However, after controlling for behavior indicators (Social Involvement of Children, Interaction Time, Response of Children to Social Cues, time for Caregiver Initiated Social interactions, Children Initiated Social interactions) and sex, relationship between verbal IQ and PSD values was not significant in any groups, which indicates that the PSD values is mainly associated with social function. This finding was different with previous researches indicating cognitive ability related to alpha power [71–73], however, most of these studies examined the performance of Alzheimer’s patients in the related cognitive paradigm rather than ASD children. Our findings of PSD of alpha and theta band provides further neural mechanism for the ability of behavioral indicators employed in this study to identify social impairments in children with ASD.

And the present study also demonstrated sex differences in the associations between EEG power and behavior indicators during caregiver-child interaction. Whereas males with ASD displayed lower theta and alpha power in the context of stronger social skills, these correlations were absent for females. In addition to the girls’ better social skills mentioned above, this may also be related to the differences in sex-specific behavior of ASD children. Research findings have identified sex differences in the way that boys and girls ASD-related behaviors which indicate that it may be easier to detect ASD behaviors in boys [59]. For example, boys with ASD have significantly more restrictive interests and repetitive behaviors than girls [74] and also exhibit greater externalizing symptomology, hyperactivity, and inattention compared to girls with ASD [75]. In the future, we may need to pay more attention to ASD girls’ characteristics and customize different interactive task for children of different sex.

There are several limitations that must be taken into consideration. First, the sample size was relatively small, larger sample sizes may be necessary to further validate the effectiveness of these screening assessments. Furthermore, the correlation trend between social function and brain activity was more pronounced in male ASD participants. This finding may be attributed to the limited number of female ASD children included in our study. Thus, future studies with a larger and more balanced sample of male and female participants could provide insight into the sex differences in social interaction patterns among children with ASD in real-world settings. Second, the present study only focused on caregiver-child interactions, rather than interactions with peers. Future research could explore the use of screening assessments and EEG indicators in peer interactions to gain a better understanding of the social difficulties in ASD children. Third, despite the use of hyper-scanning (i.e., both child and parent EEG collected), We didn’t analyze the caregivers’ EEG here because of significant artifacts. In future studies, we aim to refine our experimental design to facilitate a more comprehensive understanding of the relationship between EEG activity and behavior during social interactions between children with ASD and their caregivers. Forth, in our paper, we only concern sex and intelligence as co-variable, there are other social and economic factor may influence PSD values. We will concern more factor as co-variable in the future research. Lastly, it is important to note that our participants were exclusively Chinese school-age children aged 3–5 years. Therefore, our findings may not be generalizable to other age groups, cultures, or regions. Including participants from different age groups, countries, and regions could provide opportunities for developing new ASD screening paradigms and further validating the generalizability of our findings.

## Conclusion

In conclusion, the behavioral paradigm of caregiver-child interaction in our study has been verified as an efficient method for clinical screening young children with ASD. And the effectiveness of this approach was further validated by the examination of the PSD of alpha and theta bands. Indeed, further study with large sample, longitudinal design and multi-modal data are welcomed for the validation and embedded mechanism.

### Electronic supplementary material

Below is the link to the electronic supplementary material.

Supplementary Material 1

## Data Availability

Access to the identified participant research data must be approved by the research ethics board on a case-by-case basis, please contact the corresponding authors (feili@shsmu.edu.cn,, yefenhua@126.com ) for assistance in data access request.

## Abbreviations

ASD: autism spectrum disorder
DD: developmental delay
TD: typically developing
ROC: receiver operating characteristic
AUC: area under the receiver operating curve
P_IQ: performance Intelligence Quotient
V_IQ: verbal Intelligence Quotient
FSIQ: full scale Intelligence Quotient
ADOS-CSS: Autism Diagnostic Observation Schedule- Calibrated Severity Score
SRS: Social Responsiveness Scale
CARS: Childhood Autism Rating Scale
SI: Social Impairment
NE: Negative Emotionality
DSR: Distorted Sensory Response
SIC: Social Involvement of Children
IT: Interaction Time
RSC: Responding to Social Cues
GIS: caregivers Initiated Social interaction
CIS: children Initiated Social interaction
PSD: power spectral density
