# Supplementary material for: Caregiver-child interaction as an effective tool for identifying autism spectrum disorder: evidence from EEG analysis
Source: Child Adolesc Psychiatry Ment Health. 2023 Dec 14;17:138. doi: 10.1186/s13034-023-00690-z (PMC10722789; doi:10.1186/s13034-023-00690-z)
Supplement: Supplementary file 1 — Supplementary Material 1 [file 13034_2023_690_MOESM1_ESM.docx]

# Supplementary

**Caregiver-child dyads coding**

This coding system is designed to analyze the behavioral expressions during caregiver-child interactions in children with autism spectrum disorder. Using the ELAN software, the game videos are coded in seconds to analyze the behavioral labels of caregiver-child interactions within each second (P, PE, PR, C, CE, CR).

**The code of caregivers**

During the game, if the caregiver initiates a social interaction (including language and behavior (such as Consoling, encouraging physical touch, or expressive actions towards the child's activities that convey encouragement. For example, smiling while the child is playing, helping the child up if they fall down, or kissing the child's face)) but the child does not respond with eye contact, gestures, facial expressions, or language, it is considered as completing one social interaction. In this case, the caregiver's social initiation process is coded as "P". During the game, if the caregiver initiates a social interaction and the child responds with eye contact, gestures, facial expressions, language, or other means, it is considered as completing one social interaction. In this case, the caregiver's social initiation process is coded as "PE". Caregivers’ response refers to the caregiver's response to the child's social interaction during the game, which can be through eye contact, gestures, facial expressions, language, or other means. When the caregiver responds to the social interaction, it is coded as "PR". The response can be in the form of declarative phrases or statements that convey the same meaning as the child's verbal communication (see Table S1).

**The code of children**

Similar to the behavioral coding of caregivers, the coding of children's behavior in caregiver-child interactions is also divided into three categories: C, CE, and CR. C and CE have similar definitions to P and PE, but the social initiator is the child themselves. C indicates that the child initiates a social interaction and receives a response from the caregiver. CE indicates that the child initiates a social interaction but does not receive a response from the caregiver. CR indicates that the child responds to the social behavior of the caregiver which can be through eye contact, gestures, facial expressions, language, or other means (see Table S1).

If within one second of caregiver-child interaction, there are two or more categories of coding labels present, the priority order for coding is "CE/PE" > "C/P" > "CR/PR".

**Coding by ELAN**

We used the segmentation mode divide the whole interaction video into 1-second annotations. Two researchers meticulously interpreted the interactive content of each second by repeatedly utilizing the software's annotation replay function, following the encoding principles previously discussed. Before processing the video, researchers have passed extensive consistency training, resulting in the rate of coding annotation consistency above 85% for the same video. When the coding label of a particular annotation could not be determined, a joint discussion between the researchers was held to reach a decision. Consequently, each second of annotation had only one most suitable coding label (see Figure S1).


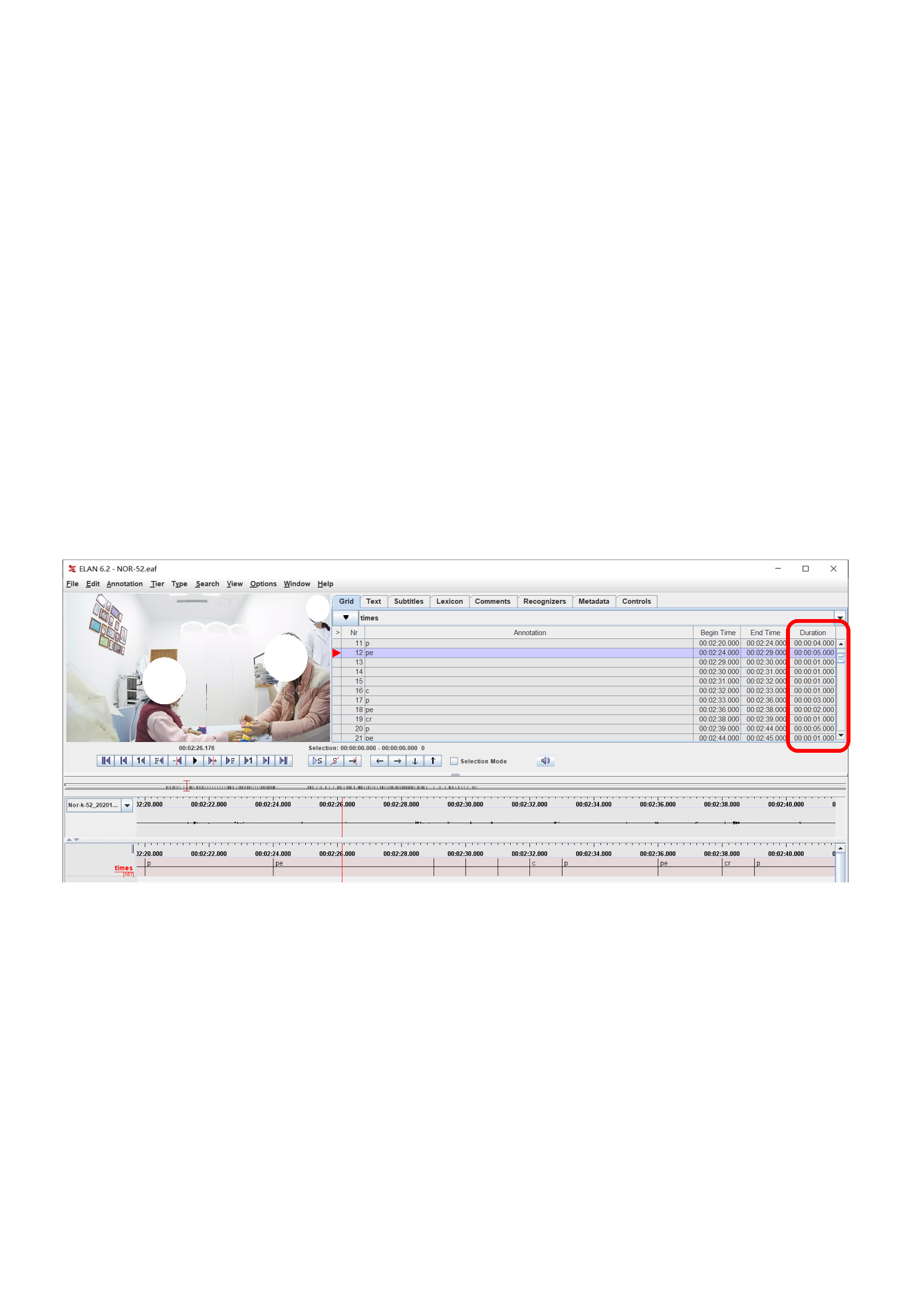


Figure S1. The ELAN interfaces.

To describe the performance of caregiver-child interactions in ASD and TD children, we used Social Involvement of Children (SIC) degree calculated as (C+CE+CR)/ (CE+C+CR+PE+P+PR), representing the percentage of time children participating in interactive games with their caregivers. The Interaction Time (IT) degree between caregivers and children, which represented the amount of time they interacted, is calculated as (PE+PR+CE+CR)/(CE+C+CR+PE+P+PR). Finally, the Response of Children to Social Cues (RSC) degree, representing the percentage of social signals initiated by caregivers that are responded to by the child, is calculated as RSC=PE/(P+PE). The proportion of time that children initiated social interactions (CIS), which represented the amount of time they initiate social interaction, is calculated as (C+CE)/(CE+C+CR+PE+P+PR). And the proportion of time that caregiver initiated social interaction (GIS), which represented the amount of time they initiate social cues, is calculated as (PE+P) / (CE+C+CR+PE+P+PR). All assessments were conducted by two experienced assessors, and the Kappa coefficient was greater than 0.7, indicating substantial agreement between the two assessors.

**Table S1. Caregiver-child coding scheme**

| **Code** | **Description** | **Example** |
| --- | --- | --- |
| C | This part of the coding system was designed to measure the proportion of child communication initiation with the caregiver but was not responded by the caregiver. |  |
| CE | It was coded when child communication initiation with the caregiver that were synchronous[1] | Child: (building) Do you like my fort? (CE)  Caregiver: It's very good. (PR) |
| CR | Child synchronous response with caregiver; Verbal or non-verbal, non-directive communication acts, comments, statements or acknowledgement, which maintain the flow and follow the caregiver's focus of attention[2]. | Caregiver: Put a red block here. (PE) Child: (puts correct block in place) (CR)OR (puts another block in place) (CR) |
| P | This part of the coding system was designed to measure the proportion of caregiver communication initiation with the child but was not responded by the child | Caregiver: Put a red block here. (P)  Child:(remains playing by himself) (NONE) |
| PE | It was coded when caregiver communication initiation with the child that were synchronous | Caregiver: Put a red block here. (PE) Child: (puts correct block in place) (CR)OR (puts another block in place) (CR) |
| PR | Caregivers’ synchronous response with child; Verbal or non-verbal, non-directive communication acts, comments, statements or acknowledgement, which maintain the flow and follow the child's focus of attention. |  |
| NONE | There was no interaction between caregivers and child during the game | Child: (Rocks a doll) (NONE)  Caregiver: That's the baby? (P)  Child: (Keep rocking) (NONE) |

**Table S2. The associations between autism symptom severity and behavior indictors within each group.**

|  | SIC  r(p) | IT  r(p) | RSC  r(p) | GIS  r(p) | CIS  r(p) |
| --- | --- | --- | --- | --- | --- |
| ASD children (with or without) group (40) | | | | | |
| CARS | -0.603(<0.001) ^a^ | -0.633(<0.001) ^a^ | -0.607(<0.001) ^a^ | 0.612(<0.001) ^a^ | -0.299(0.064) |
| ADOS-CSS | -0.013(0.936) | -0.17(0.916) | -0.067(0.685) | 0.047(0.775) | 0.047(0.777) |
| ASD children with DD group (23) | | | | | |
| CARS | -0.587(0.004) ^a^ | -0.639(0.001) ^a^ | -0.609(0.003) ^a^ | 0.586(0.004) ^a^ | -0.239(0.283) |
| ADOS-CSS | -0.491(0.020) | -0.379(0.082) | -0.368(0.092) | 0.458 (0.032) | -0.356(0.104) |
| ASD children without DD group (17) | | | | | |
| CARS | -0.664(0.005) ^a^ | -0.692(0.003) ^a^ | -0.628(0.009) | 0.701(0.002) ^a^ | -0.360(0.171) |
| ADOS-CSS | 0.238(0.375) | 0.192(0.475) | 0.267(0.318) | -0.054(0.842) | 0.470(0.66) |
| ASD children with DD group, male (19) | | | | | |
| CARS | -0.605(0.006) | -0.644(0.003) ^a^ | -0.586(0.008) | 0.615(0.005) | -0.301(0.211) |
| ADOS-CSS | -0.477(0.039) | -0.324(0.176) | -0.301(0.210) | 0.434(0.064) | -0.428(0.067) |
| ASD children without DD group, male (19) | | | | | |
| CARS | -0.664(0.005) | -0.692(0.003) ^a^ | -0.628(0.009) | 0.701(0.002) ^a^ | -0.360(0.171) |
| ADOS-CSS | 0.238(0.375) | 0.192(0.475) | 0.267(0.318) | -0.054(0.842) | 0.470(0.066) |
| Autism (with or without DD), female (5) | | | | | |
| CARS | -0.623(0.377) | -0.693(0.307) | -0.737(0.263) | 0.539(0.461) | -0.488(0.512) |
| ADOS-CSS | -0.532(0.468) | -0.595(0.405) | -0.676(0.324) | 0.438(0.562) | -0.385(0.615) |
| Autism (with or without DD), male (35) | | | | | |
| CARS | -0.547(0.001) ^a^ | 0.578(<0.001) ^a^ | -0.530(0.001) ^a^ | 0.566(0.001) ^a^ | -0.177(0.323) |
| ADOS-CSS | -0.160 (0.375) | -0.116(0.522) | -0.079(0.663) | 0.232(0.194) | -0.037(0.837) |

r presented as Pearson’s correlation coefficients, Abbreviations: PSD, Power Spectral Density; IQ, Intelligence Quotient; ASD, autism spectrum disorder; DD, developmental delay, TD, typical development.; SIC, Social involvement of the child; IT, Interaction Time; RSC, Responding to Social Cues; GIS, caregivers Initiated Social interaction; CIS, children Initiated Social interaction; ^a^, Bonferroni’s correction for multiple correlations: *p*<0.005.

1. Carter AS, Messinger DS, Stone WL, Celimli S, Nahmias AS, Yoder P: **A randomized controlled trial of Hanen's 'More Than Words' in toddlers with early autism symptoms**. *J Child Psychol Psychiatry* 2011, **52**(7):741-752.

2. Oono IP, Honey EJ, McConachie H: **Parent-mediated early intervention for young children with autism spectrum disorders (ASD)**. *Cochrane Database Syst Rev* 2013(4):Cd009774.
